# Immune Humanization of Immunodeficient Mice Using Diagnostic Bone Marrow Aspirates from Carcinoma Patients

**DOI:** 10.1371/journal.pone.0097860

**Published:** 2014-05-15

**Authors:** Melanie Werner-Klein, Judith Proske, Christian Werno, Katharina Schneider, Hans-Stefan Hofmann, Brigitte Rack, Stefan Buchholz, Roman Ganzer, Andreas Blana, Birgit Seelbach-Göbel, Ulrich Nitsche, Daniela N. Männel, Christoph A. Klein

**Affiliations:** 1 Institute of Immunology, University of Regensburg, Regensburg, Germany; 2 Project Group Personalized Tumor Therapy, Fraunhofer Institute of Toxicology and Experimental Medicine, Regensburg, Germany; 3 Experimental Medicine and Therapy Research, University of Regensburg, Regensburg, Germany; 4 Department of Thoracic Surgery, University Regensburg, Regensburg, Germany; 5 Department of Gynecology and Obstetrics, Ludwig-Maximilians-Universität München, Munich, Germany; 6 Department of Gynecology and Obstetrics, University Medical Center Regensburg, Regensburg, Germany; 7 Department of Urology, University of Leipzig, Leipzig, Germany; 8 Department of Urology, Fuerth Hospital, Fuerth, Germany; 9 Clinic of Gynecology and Obstetrics St. Hedwig, University of Regensburg, Regensburg, Germany; 10 Department of Surgery, Klinikum Rechts der Isar, Technische Universität München, Munich, Germany; B.C. Cancer Agency, Canada

## Abstract

Tumor xenografts in immunodeficient mice, while routinely used in cancer research, preclude studying interactions of immune and cancer cells or, if humanized by allogeneic immune cells, are of limited use for tumor-immunological questions. Here, we explore a novel way to generate cancer models with an autologous humanized immune system. We demonstrate that hematopoietic stem and progenitor cells (HSPCs) from bone marrow aspirates of non-metastasized carcinoma patients, which are taken at specialized centers for diagnostic purposes, can be used to generate a human immune system in NOD-scid IL2rγ(null) (NSG) and HLA-I expressing NSG mice (NSG-HLA-A2/HHD) comprising both, lymphoid and myeloid cell lineages. Using NSG-HLA-A2/HHD mice, we show that responsive and self-tolerant human T cells develop and human antigen presenting cells can activate human T cells. As critical factors we identified the low potential of bone marrow HSPCs to engraft, generally low HSPC numbers in patient-derived bone marrow samples, cryopreservation and routes of cell administration. We provide here an optimized protocol that uses a minimum number of HSPCs, preselects high-quality bone marrow samples defined by the number of initially isolated leukocytes and intra-femoral or intra-venous injection. In conclusion, the use of diagnostic bone marrow aspirates from non-metastasized carcinoma patients for the immunological humanization of immunodeficient mice is feasible and opens the chance for individualized analyses of anti-tumoral T cell responses.

## Introduction

The immunobiology of solid tumors is mostly studied in small animal models, primarily in mice. These studies are mainly based on tissue- or cell-specific expression of oncogenes, chemical-induced carcinogenesis or spontaneously arising tumors in immunocompetent mice [1]. In addition, immunodeficient mice are used to establish xenografts of human tumors, but these models suffer from either the complete absence of human immune cells or poor engraftment of certain hematopoietic lineages after hematopoietic stem and progenitor cell (HSPC) transplantation. Some authors therefore used the transfer of autologous mature peripheral blood mononuclear cells or T cells into tumor-xenograft bearing mice, but xenoreactivity of the transferred cells limits the experimental options [2]–[4].

The development of immunodeficient mice lacking the IL-2 receptor-γ chain (e.g. NOD-scid IL2rγ(null)) in the beginning of the 2000s led to a breakthrough as these new mouse strains enable an increased engraftment frequency of primary human tumor cells [5] and efficient engraftment of HSPCs with subsequent development of myeloid and lymphoid immune cells (reviewed in [6]). Simultaneous transplantation of tumor cell lines with cord-blood derived HSPCs [7] demonstrated that both cell types could successfully co-engraft. However, the impact of allograft responses against the tumor cells is unknown and consequently the experimental results may be difficult to interpret in this system. Furthermore, anti-tumoral T cell responses can’t be analyzed due to the HLA-mismatch between the tumor and human immune cells. Therefore, transplantation of syngeneic immune and cancer cells would be needed.

This may be achieved by transplantations of autologous HSPCs from peripheral blood after stem cell mobilization. Studies on healthy donors have shown long-term engraftment in immunodeficient mice [8]–. However, hematopoietic stem cell mobilization of non-metastasized carcinoma patients for research purposes is ethically questionable. Alternatively, HSPCs from carcinoma patients may be isolated from bone marrow (BM) aspirates, which are taken in specialized clinical centers for the detection of disseminated cancer cells (DCCs) following standardized diagnostic procedures [11]. Furthermore, bone marrow aspiration, when performed under aseptic conditions during general anaesthesia, is currently the preferred method to obtain hematopoietic stem cells with regard to invasiveness and morbidity. This prompted us to explore the suitability of diagnostic bone marrow aspirates from non-metastasized carcinoma patients for the generation of a patient-matched immune system in immunodeficient mice, providing a basis for preclinical tumor-immunological studies.

## Materials and Methods

### Ethics Statement

Bone marrow was aspirated from 77 unselected breast, lung, prostate or oesophageal cancer patients with no evidence of metastasis at the time of surgery and according to the standards of the International Union Against Cancer (UICC). Informed written consent was obtained according to guidelines approved by the local ethics committee (Ethics committee of the Medical Faculty of the University of Regensburg, ethics vote number 07/79).

Umbilical cord-blood was obtained from healthy, full-term pregnancies with informed written consent of the parents according to guidelines approved by the local ethics committee (Ethics committee of the Medical Faculty of the University of Regensburg, ethics vote numbers 12-101-0038 and 08/021). Peripheral mononuclear cells were isolated from adult, healthy volunteers with informed written consent according to guidelines approved by the local ethics committee (Ethics committee of the Medical Faculty of the University of Regensburg, ethics vote number 12-101-0038.) All approved experimental animal procedures were conducted to german federal and state regulations (Regierung Oberpfalz, 54-2531.1-01/08. -17/11 and -28/11).

### Mice

NOD.Cg-*Prkdc^scid^ IL2rgt^mWjl/Sz^* (also termed NSG) and NOD.Cg-*Prkdcscid Il2rgtm1Wjl* Tg(HLA-A/H2-D/B2M)1Dvs/SzJ (also termed NSG-HLA-A2/HHD) mice were purchased from the Jackson Laboratory USA and maintained under specific-pathogen free conditions, with acidified water and food ad libitum in the research animal facilities of the University of Regensburg, Germany. C57BL/6 mice were derived from in-house breedings. All approved experimental animal procedures were conducted to german federal and state regulations (Regierung Oberpfalz, 54-2531.1-01/08. -17/11 and -28/11).

### Human Cell Preparation

Mononuclear cells from human bone marrow, cord-blood and peripheral blood samples were obtained by density centrifugation on 60% Percoll (GE Healthcare, Munich, Germany), stored in RPMI 1640 o/n at 4°C, cryoconserved or immediately subjected to the isolation of CD34^+^ HSPCs. CD34^+^ HSPCs cells were isolated by positive magnetic selection using the CD34-Microbead-Kit (Miltenyi-Biotech, Bergisch-Gladbach, Germany). Purity of CD34^+^ cells was >90%. Mononuclear cells from bone marrow were cryoconserved in medium with 10% DMSO, 5% FBS, 12.5% Dextran-40 (AlleMan Pharma, Pfullingen, Germany) and 2.5% ACD-A (Sigma-Aldrich, Taufkirchen, Germany) and frozen with 1°C/min at −80°C in a cryo-freezing container (Nalgene Mr. Frosty, Sigma-Aldrich, Taufkirchen, Germany). After o/n storage at −80°C, cells were transferred to liquid nitrogen. Cells were thawed for 3 min in the waterbath at 37°C, an equal volume of medium with 2.5% BSA (Sigma-Aldrich, Taufkirchen, Germany) was added and after 10 min cells were washed with 10 ml medium plus 1.25% BSA at 300 g, 4°C for 10 min.

### Generation of Humanized Mice

Neonatal NSG mice were irradiated 24–48 h after birth with 1 Gy, adult NSG or NSG-HLA-A2/HHD mice with 3 Gy and mice were transplanted 3–5 hours later either intra-hepatically (i.h.), intra-venously (i.v.) or intra-femorally (i.f.) with CD34^+^ HSPCs. Mice were irradiated with a linear accelerator (Primus, Siemens Healthcare, Germany, 6 MV photon-emission with 2 Gy/min). Intra-hepatic injections were done with an insulin syringe (Microfine, 29G, U-50, BD Biosciences, Heidelberg, Germany) in a volume of 30 µl with up to 1×10^5^ CD34^+^ cells. For intra-femoral applications the right femur was punctured and up to 3×10^5^ CD34^+^ cells in a volume of 20 µl were injected with an insulin syringe (Microfine, 29G, U-50, BD Biosciences, Heidelberg, Germany). Mice were anesthetized i.p. for intra-femoral injections with midazolam 5 mg/kg body weight (BW), fentanyl 0.05 mg/kg BW, medetomidin 0.5 mg/kg BW. Anesthesia was antagonized with flumazenil 0.5 mg/kg BW, atipamezol 2.5 mg/kg BW, naloxon 1.2 mg/kg BW). Buprenorphin (0.1 mg/kg KG, s.c.) was injected as an analgesic. Intra-venous injections were performed into the lateral tail vain with an insulin syringe (Microfine, 29G, U-50, BD Biosciences, Heidelberg, Germany) and a volume of 200 µl with up to 4.6×10^5^ CD34^+^ cells. Morbidity associated with irradiation of 3 Gy was observed in 4,3% in NSG and in 6.5% of NSG-HLA-A2/HHD mice.

### Flow Cytometry

To validate the purity of the separated CD34^+^ cells, flow cytometric analyses were performed. Separated CD34^+^ cells were stained with FITC-conjugated anti-human hematopoietic lineage cocktail (ebioscience, Frankfurt, Germany) and PE-conjugated anti-CD34 (581, Biolegend, London, UK) for 20 min at 4°C. Afterwards cells were washed with PBS/2% FCS/0.01%NaN_3_ and fixed with 1% formaldehyde.

Levels of human hematopoietic cells in transplanted mice were assessed by multicolor flow cytometry. At defined time points, 50 µl of peripheral blood was drawn into EDTA-coated tubes and stained for flow cytometric analysis as described below. Also, at 18 to more than 20 weeks after transplantation of CD34^+^ HSPCs, spleen and bone marrow of humanized mice were removed. Single cell suspensions from bone marrow were prepared by flushing femora and tibiae. Single cell suspensions from spleen were prepared by gently grinding the organs between two layers of a 41 µm THOMAPOR strainer (Reichelt Chemie, Heidelberg, Germany). Except for peripheral blood samples of reconstituted mice, single cell suspensions of the organs were stained with viability dye eFlour 780 (ebioscience, Frankfurkt, Germany) for live/dead cell discrimination, To reduce non-specific binding single cell suspensions were incubated for 10 min at 4°C with anti-mouse CD16 (2.4G2, own production) and PBS/10% AB-serum (Bio-Rad, Munich, Germany) and subsequently stained with fluorescence-labeled antibodies for 30 min at 4°C. After lysis of erythrocytes for 10 minutes at room temperature with BD lysis solution (BD Bioscience, Heidelberg, Germany), samples were washed two times with PBS/2% FCS/0.01% NaN_3_. Cells were analyzed on a LSR II machine equipped with FACS DIVA 5.03 software (BD Bioscience, Heidelberg, Germany) and data was analyzed with FloJo 8.8.6 (Treestar, Ashland, USA). The cells were stained using the following antibodies purchased from Biolegend (London, United Kingdom): anti-human CD3-FITC (HIT3a), anti-human CD3-PerCP-Cy5.5 (SK7), anti-human CD4-PE (OKT4), anti-human CD8-Alexa Flour 700 (HIT8a), anti-human CD10-PE (MEM-78), anti-human CD19-PE (HIB19), anti-human CD33-PerCP-Cy5.5 (WM53), anti-human CD38-FITC (HIT2), anti-human CD45-Alexa Flour 700 (HI30), anti-human CD45-APC (HI30), anti-human CD45RA-Brilliant Violett 510 (HI100), anti-human CD27-Brilliant Violett 421 (O323), anti-human IgD-PerCP-Cy5.5 (IA6-2), anti-human IgM-Pacific Blue (MHM-88). The rat anti-mouse CD45-APC (30-F11) antibody was purchased from ebioscience (Frankfurt, Germany).

### Mixed Lymphocyte Reactions

Single cell suspensions from spleen and bone marrow of 2–3 mice derived from the same HSPC-donor were pooled, mononuclear cell suspensions were isolated by Percoll (65%) separation and human T cells (cT cells) were enriched with the Pan T cell Isolation Kit II (Miltenyi Biotec, Bergisch Gladbach, Germany) supplemented with a biotinylated rat anti-mouse CD45 antibody (clone 30F-11, 5 µg/ml, ebiosciences, Frankfurt, Germany) to remove mouse cells. Alternatively, human T cells (hT cells) were enriched from human peripheral blood with the Pan T cell Isolation Kit II (Miltenyi Biotec, Bergisch Gladbach, Germany). Human T cells isolated from peripheral blood or humanized mice were labeled with CFDA-SE (ebioscience, Frankfurt, Germany) at 2 µM for 10 min at 37°C in PBS/1% FBS and washed twice. CFSE-labeled T cells were cultured with irradiated cells (30 Gy) of the non-T cell fraction from the enrichment of human peripheral blood T cells or cT cells from humanized mice. Cells were plated in RPMI 1640, 2 mM glutamine, 100 U/ml penicillin, 100 mg/ml streptomycin, 10% heat-inactivated FCS, 55 µM β-mercaptoethanol (all life technologies, Darmstadt, Germany). Responder cells were plated at a concentration of 2×10^5^ per well with or without stimulator cells (2–4×10^5^/well) in round-bottom 96-well tissue culture plates in a total volume of 200 µl at 37°C and 5% CO_2_. After 5 or 6 days cells were harvested and stained with anti-human CD3-PerCP-Cy5.5 and anti-human CD45-APC and the proliferation of CD45^+^CD3^+^ cells was analyzed by flow cytometry.

Alternatively, BM-derived DCs from the BM of femura and tibiae of NSG-HLA-A2/HHD mice were generated. Cells were cultured in RPMI 1640 supplemented with 2 mM glutamine, 100 U/ml penicillin, 100 mg/ml streptomycin, 10% heat-inactivated FCS and 1% v/v murine granulocyte–macrophage colony-stimulating factor (GM-CSF, own production) as described previously [12]. Cells were harvested at day 8 and stimulated overnight with 1 mg/ml LPS (E.coli O127:B8, Sigma-Aldrich, Taufkirchen, Germany). After washing cells for 3 times in PBS, cells were used as stimulator cells at a concentration of 0.05–3×10^4^ cells per well with 1×10^5^ murine CD8 T cells (C57BL/6) or human peripheral blood CD8 T cells as responder cells.

### Statistical Analysis

Statistical analysis was done using GraphPad Prism 6.0 software (GraphPad Software, Inc., San Diego, USA). Differences in medians between groups were analyzed by Student’s t-test, Mann-Whitney-U or Kruskal-Wallis test where appropriate. Correlations were evaluated using the Spearman rank correlation coefficient method. A p-value of less than 0.05 and 0.001 were considered to be statistically significant and highly significant, respectively.

## Results

**Figure 1:**
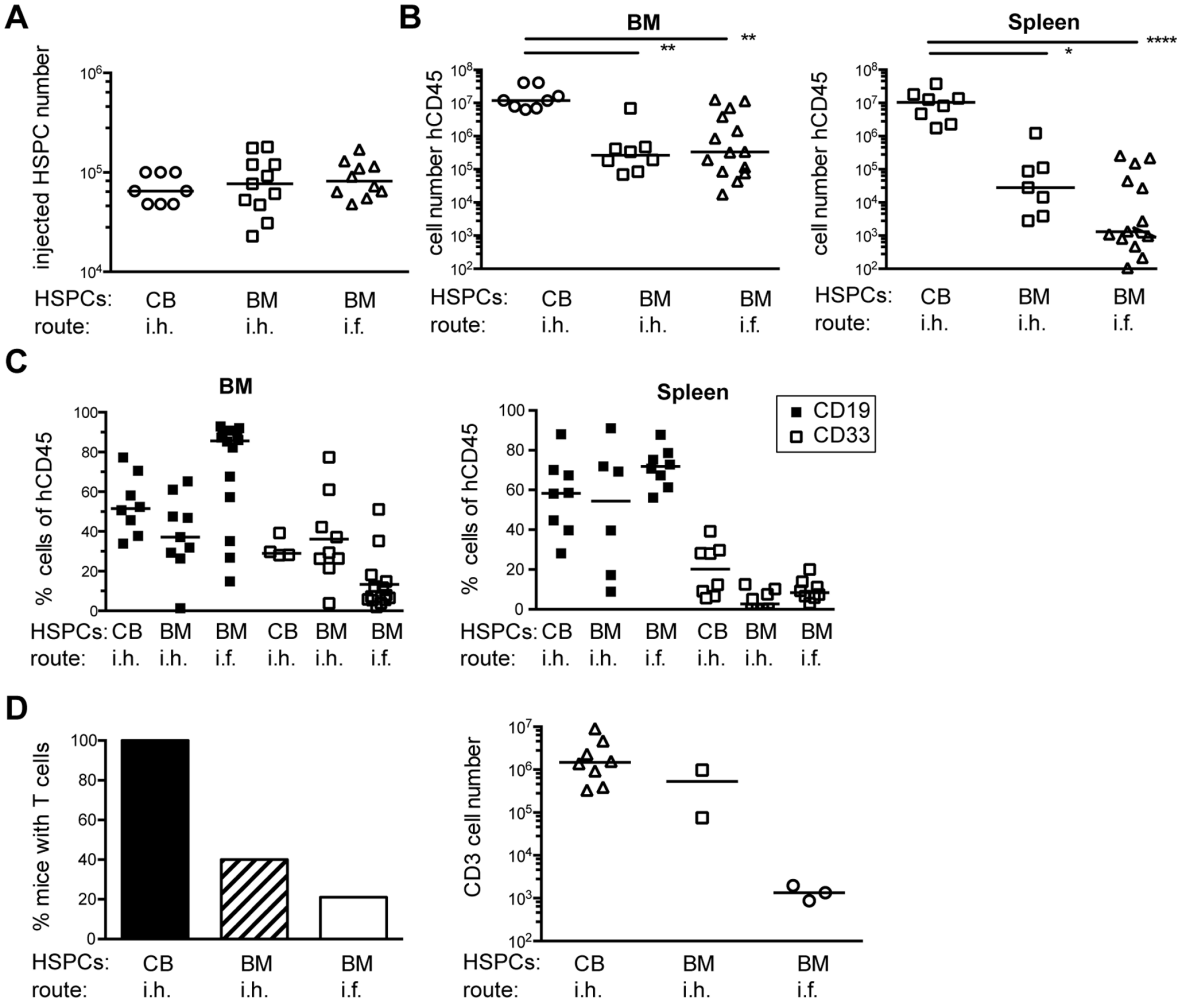
HSPC engraftment in NSG-mice by intra-femoral or intra-hepatic injections. Adult or neonatal NSG mice were sublethally irradiated and transplanted either intra-hepatically (neonatals: CB-HSPCs n = 8, 3 donors; BM-HSPCs n = 11 donors) or intra-femorally (adult: BM-HSPCs n = 14, 11 donors) with CD34^+^ HSPCs. Mice were analyzed at >20 weeks after transplantation by flow cytometry. (**A**) The number of injected CD34^+^ HSPCs for each group is given. (**B**) Engraftment was determined by human CD45 expression. The absolute number of hCD45^+^ cells in BM and spleen of reconstituted mice is shown. Indicated are values for significant differences between the groups. Bone marrow: CB i.h. vs. BM i.h., p = 0.003; CB i.h. vs. BM i.f., p = 0.004; Spleen: CB i.h. vs. BM i.h., p = 0.04; CB i.h. vs. BM i.f., p<0.0001; ANOVA, Kruskal-Wallis). Each symbol represents an individual animal. (**C**) The proportion of B cells and myeloid cells in BM and spleen is shown as percentage of human CD45^+^ cells. Each symbol represents an individual animal. (**D**) left: The percentage of mice with T cells in spleen at >20 weeks after transplantation is given. right: The absolute number of CD3^+^ T cells in the spleen of mice with T cells is shown. Due to the limited number of samples in two of the three groups no statistically analysis was performed.

Diagnostic bone marrow aspirates were obtained from 77 non-metastasized carcinoma patients diagnosed with non-metastasized lung (n = 29), breast (n = 28), prostate (n = 14) or oesophageal cancer (n = 6), and with a median age of 62 years (Table 1). The aspirates were obtained from five different clinical sites during primary tumor resection of the patients. To achieve long-term reconstitution of NSG mice with human immune cells, an intra-hepatic injection of CD34^+^ HSPC into newborn NSG mice is currently considered to result in best engraftment [13]. As the arrival of diagnostic bone marrow aspirates cannot be scheduled and rarely matches with the availability of newborn mice, we tested whether intra-femoral injections of BM-derived CD34^+^ HSPC into adult NSG mice gives a comparable reconstitution with human immune cells as intra-hepatic injections of BM- or cord-blood derived HSPCs into neonatal mice. An intra-femoral injection is considered as an appropriate alternative route for the transplantation of a limited number of human HSPCs [14]. Hence, we cryoconserved cord-blood (CB) or bone marrow mononuclear cells (BM-MNCs), isolated HSPCs on the day of transplantation and injected all mice with comparable numbers of cells (Figure 1A). Depending on their age, neonatal and adult mice of the intra-hepatic and intra-femoral groups were irradiated with different doses of sublethal irradiation (1 vs. 3 Gy), which might affect stromal cell numbers differently. We therefore compared the reconstitution level between the groups by the absolute numbers and not the percentage of human CD45 cells in BM and spleen (Figure 1B).

**Table 1:** Patient data.

	statistics	sample volume [ml]	BM-MNCs [×10^6^]	HSPC [×10^6^]	HSPCs per 10^6^ BM-MNCs [×10^4^]	age [years]
**BM (n = 77)**	**median**	15.3	71	0.8	5.3	62
	**min-max**	5–27	11.8–1090	0.01–110	0.06–47.9	30–83
**cord-blood (n = 31)**	**median**	60	275	1.1	1.6	–
	**min-max**	30–100	73–1300	0.17–3.36	0.3–4.2	–

For human bone marrow aspirates of non-metastasized carcinoma patients and cord-blood samples, the median sample size, age and obtained number of BM-MNCs and HSPCs are given. The distribution of the carcinoma type was as follows: lung 29/77, mammary 28/77, prostate 14/77 and oesophageal 6/77.

The reconstitution with human CD45 cells in BM and spleen of mice that had received cord-blood derived HSPCs was highest of all groups, consistent with the known better immune reconstitution obtained with HSPCs from neonatal than adult donors (Figure 1B) [15]–[17]. In contrast, mice transplanted with BM-HSPCs intra-hepatically or intra-femorally displayed fewer human leukocytes, with no significant difference between both groups. Analyzing the contribution of the myeloid and lymphoid lineage to the immune reconstitution, we found that mice transplanted with BM-HSPCs developed myeloid and B cells (Figure 1C), but only 20–40% of the mice had detectable T cells in the spleen 20 weeks after transplantation (Figure 1D). In contrast, all mice transplanted with CB-HSPCs developed T cells and had higher numbers of T cell. Due to the low number of transplanted mice that developed T cells no definite conclusion can be drawn regarding the best application route of cryopreserved BM-HSPCs (Fig. 1D).

**Figure 2:**
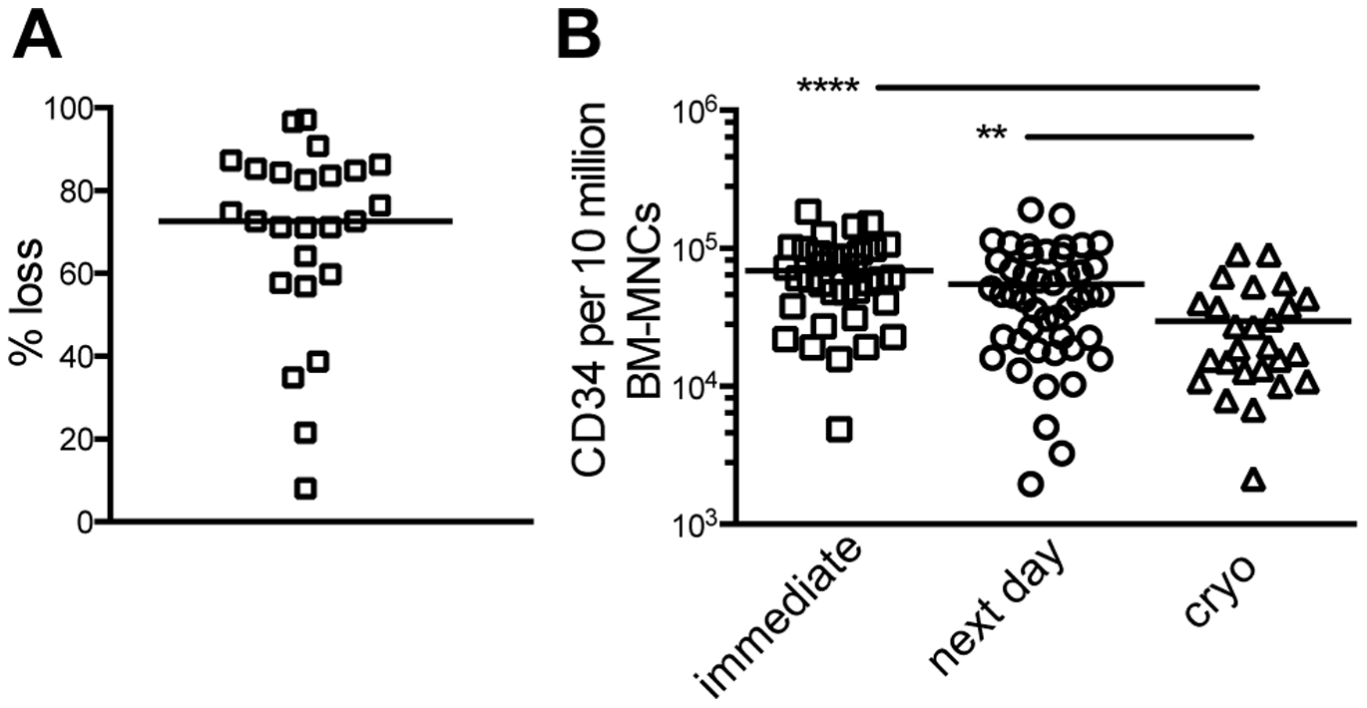
Yield of CD34^+^ HSPCs isolated from cryoconserved and non-cryoconserved BM- MNCs. (**A**) BM mononuclear cells from BM-aspirates were isolated by density centrifugation and frozen in liquid nitrogen. After maximally 6 months of storage cells were thawed and counted. (**B**) CD34^+^ HSPCs were isolated from BM-MNCs at the day of arrival (immediate), the next day after overnight storage of BM-MNCs at 4°C or from cryoconserved samples. The number of CD34^+^ cells per 10 million BM-MNCs is shown (p = 0.001 immediate vs. cryo and p = 0.0168 next day vs. cryo; ANOVA Kruskal-Wallis).

In line with these data, we had observed that thawing of BM-MNCs resulted in a median loss in the sample’s cellularity of 72% (8%–85%, Figure 2A). Therefore, we examined the effect of cryoconservation of BM-MNCs on the yield of CD34^+^ cells and the qualitative and quantitative immune cell reconstitution. For this, HSPCs were isolated either on the day of arrival or the next morning after overnight storage of BM-MNCs at 4°C. These samples were compared to samples of which BM-MNCs were cryoconserved on the day of arrival and thawed shortly before HSPC-isolation and transplantation.

**Figure 3:**
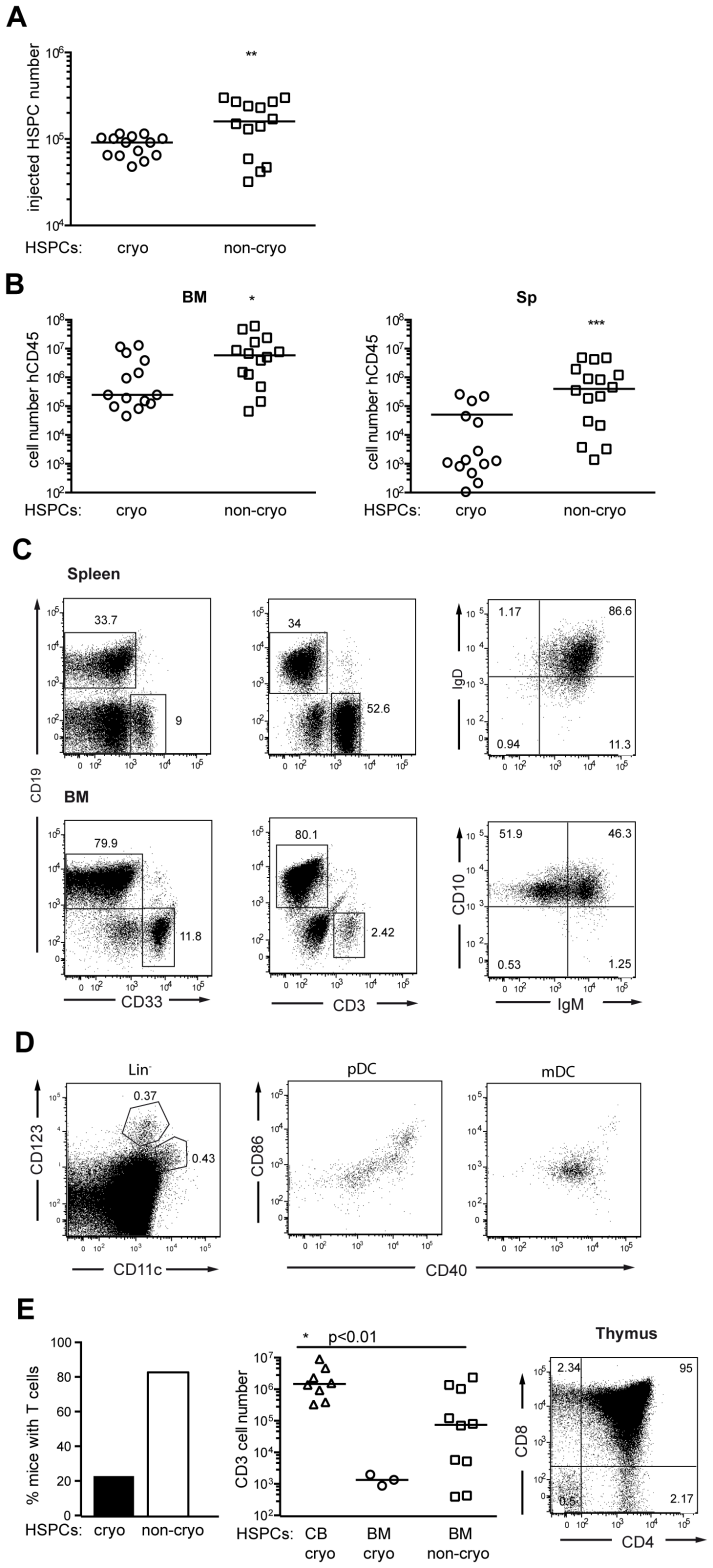
NSG mice engrafted with cryoconserved or non-cryoconserved BM-HSPCs by intra-femoral injections. Adult NSG mice were sublethally irradiated and transplanted with cryoconserved (n = 14, 11 donors) or non-cryoconserved BM-HSPCs (n = 14, 13 donors). At >20 weeks after transplantation mice were analyzed by flow cytometry. (**A**) The number of injected CD34^+^ HSPCs for each group is given. As the yield of CD34^+^ cells is higher from non-cryoconserved BM samples, the respective group received significantly more HSPCs (p = 0.0045, student‘s t-test). (**B**) Engraftment in BM and spleen was determined by human CD45 expression. The absolute number of hCD45^+^ cells in BM and spleen of the reconstituted mice is shown, each symbol represents an individual animal. Indicated are values for significant differences between the groups (BM: p = 0.03, Spleen p = 0.0003, Mann-Whitney test) (**C**) Representative flow cytometric analysis of BM and spleen for CD19^+^ B cells, CD33^+^ myeloid cells and CD3^+^ or CD3^+^CD4^+^/CD8^+^ T cells. B cell developmental stages in BM and spleen are shown for CD24^+^CD38^high/low^ cells. In BM pro/pre-B cells were defined as CD10^+^ IgM^-^, immature B cells as CD10^+^IgM^+^. In spleen mainly transitional (IgD^+^IgM^+^) B cells were detected. (**D**) Representative flow cytometric analysis of the spleen for plasmacytoid and myeloid dendritic cellls (pDC: Lin^-^CD123^+^CD11c^-^; mDC: Lin^-^CD123^−^CD11c^+^) and their expression of costimulatory molecules (CD40, CD86) (**E**) Representative flow cytometric analysis of CD3^+^ T cells in the thymus and their expression of CD4 and CD8 at >20 weeks after transplantation. The percentage of mice with CD3^+^ T cells and their absolute number in spleen at >20 weeks after transplantation is given (CB vs. non-cryo BM: p<0.01, Mann-Whitney test).

A significantly lower yield (determined as the number of CD34^+^ cells per 10 million BM-MNCs) was obtained when HSPCs were isolated from cryoconserved samples (immediate vs. cryo: p = 0.001; next day vs. cryo: p = 0.017; Figure 2B). In contrast, no significant difference (p = 0.27) could be detected in the yield of HSPCs between samples processed on the day of arrival (immediate) or after overnight storage at 4°C (next day) (Figure 2B). Next, we transplanted intra-femorally HSPCs from cryoconserved or non-cryoconserved BM samples (Figure 3A). We used from all samples the maximal number of HSPCs as our interest was to identify the most suitable protocol. This resulted in injection of a higher median number of HSPCs from non-cryoconserved samples (p = 0.005). For bone marrow and spleen we found no significant difference (both p = 0.21) in the reconstitution with hCD45^+^ cell for immediately isolated and transplanted BM-HSPCs *vs.* samples injected on the next day after overnight storage of BM-MNCs at 4°C (Figure S1). Therefore, we combined these samples as non-cryoconserved group and compared it to the group transplanted with cryoconserved cells.

We observed a significantly lower reconstitution with hCD45^+^ cells in BM and spleen, if HSPCs were isolated from cryoconserved BM (BM: p = 0.03; Spleen: p = 0.0003, Mann-Whitney test; Figure 3B). Comparing the development of the different hematopoietic cell lineages, we found that both groups developed myeloid cells and B cells (Figure 3C). Consistent with previous reports, the majority of B cells did not develop beyond the immature/transitional B cell stage in BM and spleen [18]. In spleen myeloid dendritic cells and plasmacytoid dendritic cells were detected, with the latter showing upregulation of CD86 and CD40 (Figure 3D). Interestingly, the percentage of mice with T cell development in the spleen was clearly increased (83.3% vs. 21%), in the group with non-cryoconserved HSPCs and almost reached the level obtained with intra-hepatic transplantations of cord-blood derived HSPCs (Figure 3E). Yet, the splenic T cell number in mice reconstituted with non-cryoconserved BM-HSPCs was significantly lower than in CB-HSPC transplanted mice (p<0.01; Mann-Whitney test). Nevertheless, we could observe T cell development in the thymus more than 20 weeks after transplantation of BM-HSPCs.

In the thymus epithelial cells mediate positive selection of developing T cells. As NSG mice do not express HLA-molecules on thymic epithelial cells, human T cells developing in these mice lack the ability to recognize antigens and function in an HLA-restricted manner [6], [19]. In NSG-HLA-A2/HHD mice [19] the HLA-A2 allele is ubiquitously expressed, which prompted us to test whether the inferior T cell development in BM-HSPC reconstituted NSG-mice could be improved in HLA-I transgenic NSG-mice.

**Figure 4:**
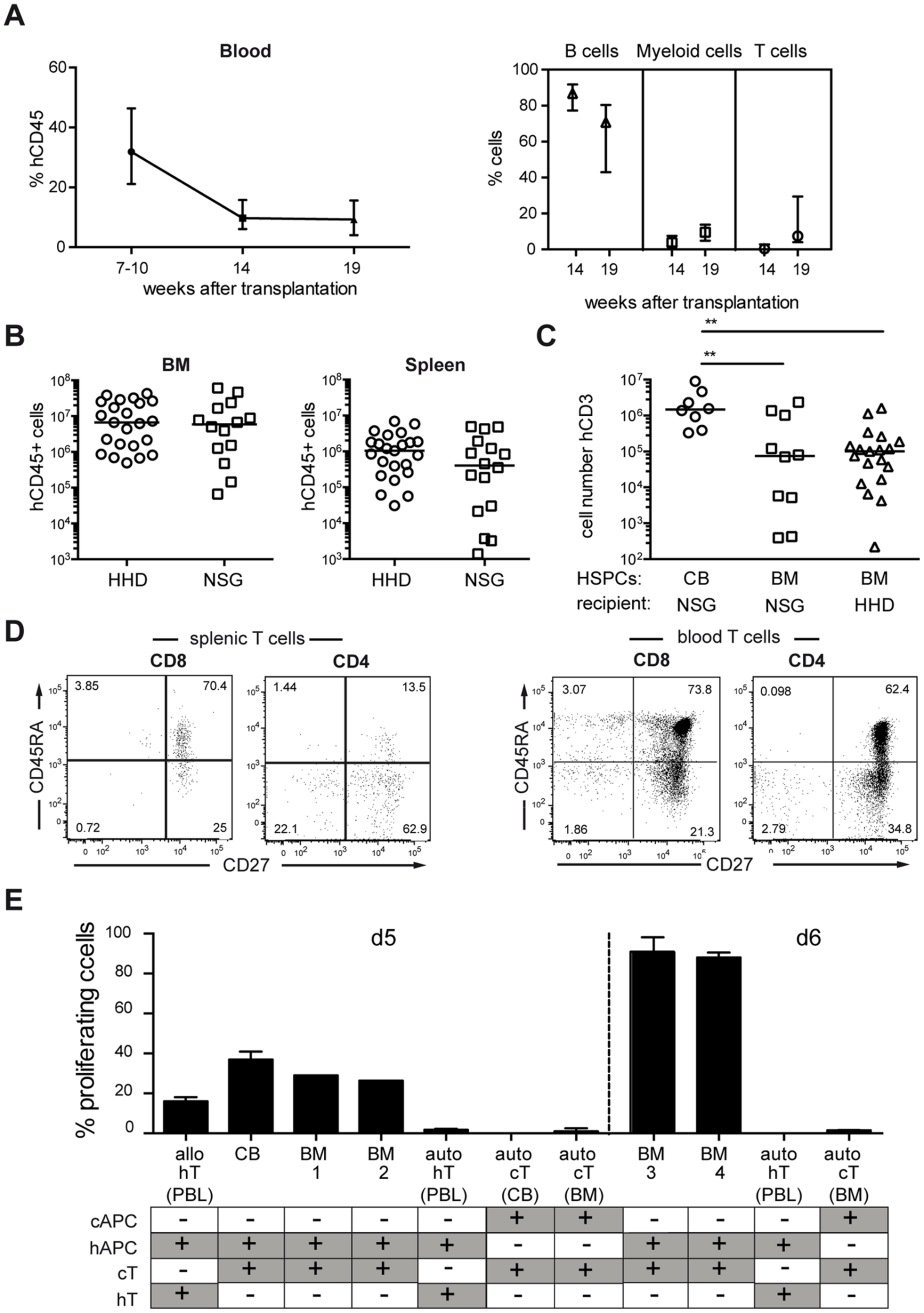
Reconstitution of a functional, HLA-restricted and self-tolerant T cell repertoire. Adult sublethally irradiated NSG-HLA-A2/HHD mice were transplanted intra-venously with cryoconserved CD34^+^ HSPCs (n = 23 mice, 13 donors). At different time points after transplantation mice were analyzed by flow cytometry and final analysis of organs was performed at 18–20 weeks after reconstitution. (**A**) Flow cytometric analysis of peripheral blood of reconstituted mice (n = 12 mice) at 7–19 weeks after BM-HSPC transplantation. Shown is the % of hCD45^+^, hCD45^+^CD19^+^ (B cells), hCD45^+^CD33^+^ (myeloid cells) or hCD45^+^CD3^+ (^T cells) cells (median with interquartile range). (**B**) Engraftment in BM and spleen was determined by human CD45 expression. The absolute number of hCD45^+^ cells in BM and spleen of the reconstituted mice is shown and compared to NSG-mice reconstituted with non-cryoconserved BM-HSPCs (see Fig. 3B). Each symbol represents an individual animal. Indicated are values for significant differences between the groups (BM: p = 0.64; Spleen: p = 0.19, Mann-Whitney test). (**C**) In comparison to CB- (n = 8 mice) and BM-HSPC (n = 10 mice) transplanted NSG mice (i.h. and i.f. respectively), the absolute number of splenic CD3^+^ T cells is given for NSG-HLA-A2/HHD mice (n = 19 mice). Each symbol represents an individual animal. Indicated are values for significant differences between the groups (CB vs BM-NSG: p = 0.009; CB vs BM-HHD: p = 0.0027, Kruskal-Wallis test). (**D**) Representative flow cytometric analysis of splenic CD3^+^CD4^+^ and CD3^+^CD8^+^ T cells and their respective expression of CD27 and CD45RA. In addition, CD27 and CD45RA expression of peripheral blood T cells of a healthy volunteer is shown. (**E**) Mixed lymphocyte reaction of human T cells isolated from NSG-HLA-A2/HHD mice ( = chimeric T cells, cT) transplanted with CB- (n = 1 donor, 2 mice) or BM-HSPCs (n = 4 donors, each 2–3 mice). CFSE-labeled cT cells were stimulated with human T cell-depleted and irradiated peripheral blood mononuclear cells (hAPC, d5∶2×10^5^ cells/well; d6∶2×10^5^ cells/well). As control, human T cells from peripheral blood of the same (auto, n = 2) or different (alllo, n = 2) HLA-A2 negative donor were isolated. Self-tolerance was tested incubating cT cells or hT cells on autologous T cell depleted, irradiated cAPC or hAPC from the same humanized mouse or human volunteer. Proliferation of T cells was measured after 5 or 6 days based on the gradual loss of the CFSE-label.

Intra-femoral transplantations in comparison to intra-venous injections are technically difficult and harbor the risk of lethal infections for the recipient mice. Furthermore, the most commonly used injection routes for HSPC into neonatal NSG mice (i.h., i.v., intra-cardiac) all introduce the injected cells directly into the circulation and no difference in engraftment was observed [13]. We therefore injected the BM-HSPCs intra-venously into NSG-HLA-A2/HHD mice. Analysis of the peripheral blood at 7, 10, 14 and 19 weeks after HSPC-transplantation revealed that the percentage of hCD45 cells decreases over time, but stabilizes around week 14 (Fig. 4A) suggesting that at around this time point immune cell reconstitution starts to depend on HSC with long-term reconstitution potential. The percentage of T cells in blood started to increase after week 14 of HSPC-transplantation.

As reported for CB-HSPCs [19], NSG-HLA-A2/HHD mice did not show a significant difference in the overall immune cell reconstitution in BM and spleen as compared to NSG mice (Fig. 4B). Interestingly, even though we transplanted NSG-HLA-A2/HHD mice with cryoconserved HSPCs, 82% (19/23) of the mice showed T cell development 19–20 weeks after transplantation. However, the absolute number of CD3 cells was still lower than that of neonatal NSG mice transplanted with CB-HSPCs and no significant difference in the number of CD3, CD4 (not shown) or CD8 T cells (not shown) in the spleen of NSG-HLA-A2/HHD and NSG-mice was observed (Figure 4C).

Although we could not observe any superiority of NSG-HLA-A2/HHD over NSG-mice with regard to overall engraftment and T cell development, we further explored this mouse strain, as these mice were recently shown to enable HLA-I restricted T cell responses after transplantation of human CB-HSPC [19] and should enable the investigation of human CD8 T cell responses against human, autologous cancers of HLA-A2^+^ patients. As a first glimpse into T cell and DC function, we investigated their ability to respond and activate. CD8 T cells in the spleen of NSG-HLA-A2/HHD mice displayed a similar naive (CD45RA^+^CD27^+^) phenotype as CD8 T cells from human peripheral blood, whereas the majority of CD4 T cells showed signs of antigen-experience (CD45RA^-^CD27^+/−^, Figure 4D). To analyze whether functional and self-tolerant, human T cells had developed from BM-HSPCs, we subjected these T cells to an *in vitro* mixed lymphocyte reaction using allogeneic and autologous stimulator cells (Figure 4E). To that end, we isolated human T cells from spleen and bone marrow of mice humanized with BM-HSPCs from four different donors or from mice transplanted with CB-HSPCs and referred to these cells as chimeric T cells (cT cells). The cT cells were stimulated with human T cell-depleted and irradiated mononuclear cells (hAPC) from peripheral blood of a healthy HLA-A2 negative volunteer. As control, hT cells from the same (auto) or different (allo) donor were isolated and stimulated with hAPC. To test for self-tolerance, cT cells isolated from cord-blood or BM-HSPC reconstituted mice were cultured with autologous cAPC isolated from the same humanized mouse, whereas hT cells were stimulated with autologous hAPC. On day 5 of the mixed lymphocyte reaction, proliferation of cT cells from both, CB- and BM-HSPC transplanted mice could be detected when incubated with allogeneic hAPC. The percentage of alloreactive cT cells from BM-HSPC reconstituted mice was similar to cT cells from CB-HSPCs transplanted mice. When the number of stimulator cells (hAPC) was doubled and proliferation analyzed on day 6, ≥90% human T cells from BM-HSPC transplanted mice proliferated. Importantly and identical to hT cells stimulated with autologous hAPC, cT cells from BM- or CB-HSPC transplanted mice were self-tolerant and did not proliferate on autologous cAPC (auto, d5+ d6). These cAPC were functional stimulator cells as they could induce proliferation of allogeneic hT cells in a dose-dependent manner (Figure S3A). Theoretically, also murine HLA-A2 expressing APCs could have activated hT cells in this assay. Therefore, we compared the ability of *in vitro* generated BM-derived, LPS-stimulated and HLA-A2 expressing murine DCs from non-reconstituted NSG-HLA-A2/HHD mice to activate human allogeneic CD8 T cells from a HLA-A2 negative donor or murine C57BL/6-derived CD8 T cells in an *in vitro* mixed-lymphocyte reaction (Figure S3B). Whereas NSG-HLA-A2/HHD BM-DCs could induce proliferation in up to 37% of the murine CD8 T cells, only maximally 5% of the human CD8 T cells were dividing. These findings suggest that the activation of hT cells by cAPCs can be mainly attributed to human antigen-presenting cells reconstituted from transplanted BM-HSPCs.

These experiments assessed the influence of several factors (cryoconservation, HLA-A2 expression, age of recipient mice at transplantation, method of cell injection) on the immune cell reconstitution and addressed the immune system’s ability to mount a robust immune response (i.e. an allograft response). We then sought to determine the number of BM-HSPCs required to achieve CB-equivalent reconstitution. Finally, we asked whether this number of HSPCs could be obtained from a single diagnostic bone marrow to reconstitute several mice.

**Figure 5:**
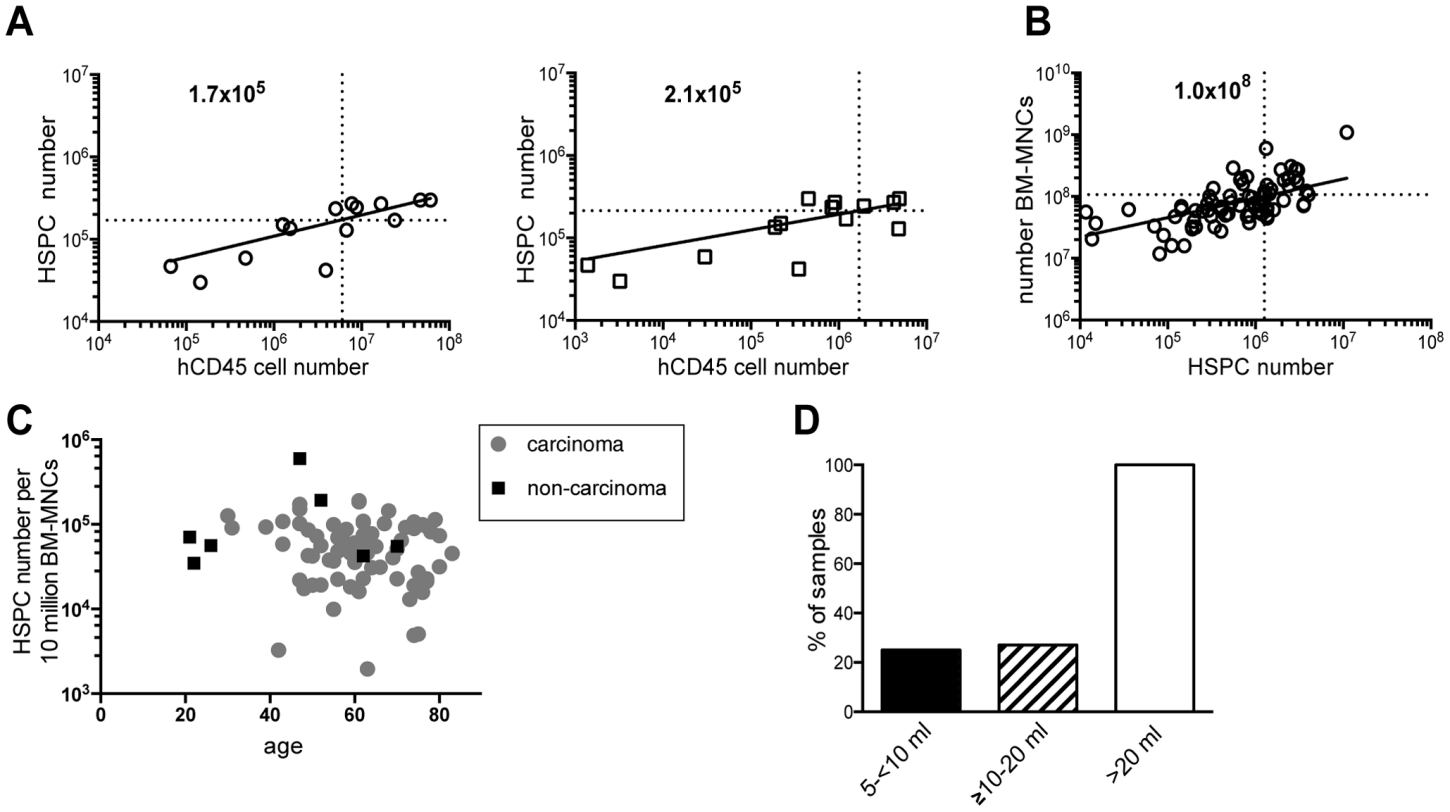
Determination of the minimal number of BM-HSPCs, BM-MNCs and volume of BM-aspirate required for the reconstitution of NSG mice. Six-to eight week-old sublethally irradiated NSG mice were transplanted intra-femorally with non-cryoconserved CD34^+^ HSPCs (n = 14, 11 donors). (**A**) At >20 weeks after transplantation mice were analyzed by flow cytometry and engraftment in BM and spleen was determined by human CD45 expression and plotted vs. the number of injected HSPCs. The number of BM-HSPCs is calculated, which is required to achieve reconstitution in BM and spleen comparable to the lowest number of transplanted CB-HSPCs (4.8×10^4^ injected CD34^+^ HSPCs) that gives a reconstitution level matching with published data (BM: 6×10^6^ hCD45^+^; spleen 1.7×10^6^ hCD45^+^). Linear regression analysis revealed that at least 1.7–2.1×10^5^ BM-HSPCs are needed. (**B**) The number of BM-MNCs at sample receipt is plotted vs. the number of isolated HSPCs. The number of BM-MNCs (10^8^) is calculated that is required to isolate HSPCs sufficient for the reconstitution of six mice from one BM sample, given that each mouse receives 2.1×10^5^ HSPCs. (**C**) The number of CD34^+^ HSPCs per 10 million BM-MNCs is plotted vs. the age of carcinoma (n = 77) or control-patients (n = 7). No correlation is observed. (**D**) Percentage of samples and the volume of samples at receipt, which contained ≥10^8^ BM-MNCs allowing the isolation of HSPCs sufficient for the transplantation of six mice.

We used the group of NSG mice transplanted with non-cryoconserved BM-HSPCs for this analysis. We defined the CB-equivalent reconstitution as the number of human leukocytes in spleen and bone marrow that was obtained with the lowest number of transplanted CB-HSPCs in our study that gives a reconstitution level matching with published data [20]: the transplantation of 4.8×10^4^ CB-HSPCs resulted in 1.7×10^6^ and 6×10^6^ human leukocytes in spleen and bone marrow, respectively. In BM-HSPC humanized mice, a highly significant correlation was found between the number of transplanted HSPCs and human leukocytes in bone marrow and spleen of humanized mice (BM: r = 0.85, p = 0.0002; Spleen: r = 0.67, p = 0.001; Spearman). Linear regression analysis (Figure 5A) showed that about 1.7–2.1×10^5^ CD34^+^ cells, i.e. approximately 5-fold more BM- than CB-HSPCs, are needed to reach CB-equivalent reconstitution.

Usually, less than 10 animals can be reconstituted with one cord-blood sample [21], whereas from bone marrow aspirates of healthy volunteers and type I diabetes patients 1–2 and 7 recipient mice were generated, respectively [16], [22]. We therefore calculated the number of BM-MNCs required for the isolation of 1.26×10^6^ HSPCs, a number sufficient for the generation of six mice with CB-equivalent reconstitution level as stated above. This number of mice would enable meaningful experimental comparisons from one patient. The number of isolated HSPCs from non-cryoconserved samples correlated with the number of patient-isolated BM-MNCs (r = 0.66; p<0.0001; Spearman) and linear regression analysis revealed that 1×10^8^ BM-MNCs (Figure 5B) are required for the isolation of 1.26×10^6^ HSPCs.

For assessment of minimal systemic cancer spread to bone marrow, we had obtained diagnostic bone marrow aspirates of 77 M0-stage patients. We received a median volume of 15.3 ml and could isolate in median 7.1×10^7^ BM-MNCs (Table 1). From these 77 aspirates, 28 samples (36%) had ≥10^8^ BM-MNCs and from 31 samples (40%) ≥10^6^ HSPCs could be isolated. We did not observe a correlation between the number of isolated CD34^+^ cells and patient age (Figure 5C) or carcinoma type (Figure S2). Also, the frequency of CD34^+^ HSPCs in carcinoma patients was similar to non-carcinoma patients (Figure 5C). Interestingly, the number of BM-MNCs allowed a better estimation whether ≥10^6^ HSPCs would be obtained than the volume of the arriving bone marrow aspirate: the chance to isolate 10^6^ HSPCs cells differed marginally between samples with 5–10 ml or 10–20 ml volume, i.e. a 5 ml aspirate could contain a similar number of isolated HSPCs than a 20 ml sample (Figure 5D). Only when we had received more than 20 ml bone marrow we could always isolate the required number of HSPCs.

## Discussion

Here we show that an immune system can be generated in immunodeficient mice using CD34^+^ HSPCs from bone marrow aspirates of non-metastasized carcinoma patients. Bone marrow sampling is performed to assess minimal cancer spread at the time of primary surgery and the prognostic value of DCC detection in bone marrow is currently considered to be better than that of circulating tumor cells [23], [24]. Therefore, bone marrow is, in addition to the primary tumor, a source of highly relevant cancer cells whose interaction with immune cells is unstudied so far. This limitation could be overcome if autologous cancer and immune cells were explored in double-humanized mouse models. The specific challenge of such experiments lies in the complex interdependence of sample and recipient mouse availability with experimental needs and conditions. Here, we analyzed the requirements for immune humanization such that it can be combined with autologous xenografts from bone marrow, blood, or primary tumor derived cancer cells in the future.

We used cord blood HSPCs as gold standard and compared sample preparation methods and routes of HSPC application. We observed that cryoconservation of BM-MNCs negatively affects the yield of isolated CD34^+^ HSPCs and reconstitution with hCD45^+^ in BM and spleen, which was particularly evident for peripheral T cells. This is in line with a recent study showing that cryoconservation of cord-blood cells not only decreases the percentage of peripheral blood hCD45^+^ cells, but also the proliferation of T cells *in vitro* in response to anti-CD3 stimulation [25]. In contrast, overnight storage of BM-MNCs with isolation and transplantation of HSPCs on the next day, neither affects the HSPC-yield, nor the *in vivo* immune cell reconstitution. Given the significant drop of total cell recovery upon thawing of BM-MNCs, it is most likely that the number of long-term hematopoietic repopulating cells is also greatly diminished. We found that five-times more BM-HSPCs need to be injected to achieve reconstitution indices comparable to CB-HSPCs. This is consistent with the previously discussed reduced repopulating and lymphopoietic activity of BM-HSPCs compared to CB-HSPCs [15], [16], [26]. Therefore, reduced stem cell numbers in cryoconserved samples will have greater impact on the reconstitution with BM-samples than CB-samples.

The sample receipt of diagnostic bone marrow rarely matches the availability of newborn mice. This fact and the negative impact of freeze-thaw cycles necessitate transplanting HSPCs into adult mice. We found that the qualitative and quantitative immune cell reconstitution does not differ significantly between intra-femoral injections into adult and intra-hepatic injections into neonatal mice of BM-HSPCs. In addition, we did not observe disadvantages from intra-venous injections for CB-HSPCs when compared to intra-femoral and intra-hepatic injections (unpublished data). Also, the development of functional T cells in NSG-HLA-A2/HHD mice after i.v. injection shown here suggests that this is not different for BM-HSPCs. Therefore, although we did not directly compare intra-femoral and intra-hepatic with intra-venous injections, we expect i.v. injections to result in comparable engraftment. As i.v. injections are easy and fast to perform and carry minimal risk of infection for the severely immunocompromised recipients, we now prefer intra-venous injections into adult mice for the transplantation of BM-HSPCs.

We deemed particularly interesting to assess whether the human antigen-presenting cells and T cells that are reconstituted from BM-HSPCs can mold an allogeneic T cell response. To this end we tested and confirmed both, self-tolerance and reactivity of T cells developing in NSG-HLA-A2/HHD mice, and the potential of cAPCs to prime T cell responses. Of interest, the proportion of mice with long-term T cell reconstitution in spleen was higher in NSG-HLA-A2/HHD than in NSG mice, although we used cryoconserved BM-HSPCs for this experiment. Therefore, it is very likely that T cell development in NSG-HLA-A2/HHD mice may be improved by using freshly isolated BM-HSPCs or transplanting into younger recipient-mice (3–4 weeks instead of 6–8 weeks) before thymic involution [13]. Additional enhancement may be achieved by supplementing homeostatic cytokines such as hIL-7 [27], as murine IL-7 has only low activity on the human receptors [28]. A more difficult way employs additional transplantation of HLA-matched fetal thymic grafts [22], as this requires the availability of a ready-to-use bank of fetal-thymic tissue, HLA-matched for at least one allele of the cancer patient.

Finally, we retrospectively analyzed how diagnostic bone marrow samples should be selected to set up double-humanized mice, consisting of autologous immune and cancer cells. We found that about 2.1×10^5^ HSPCs per animal are needed to achieve a CB-equivalent reconstitution. Since six times this number can be obtained from a BM volume of 10–20 ml (the volume recommended by the consensus protocol for DCC detection [11]), in 40% of cases about six mice can be generated from one sample. This number is in the range of previous experiments using adult bone marrow [22]. Slightly higher aspirate-volumes would enable reconstitution of more animals and increase the proportion of suitable samples. Alternatively, recently developed protocols for the *in vitro* expansion of long-term repopulating cord-blood hematopoietic stem cells [29], [30] might prove helpful for BM hematopoietic stem cells as well. Taken together, we provide an important framework for the use of diagnostic BM-aspirates from carcinoma patients as adult HSPC-source. Future experiments need to assess whether double-humanized NSG-HLA-A2/HHD mice from BM-HSPCs and autologous cancer cells of HLA-A2^+^ patients will generate HLA-A2 restricted T cell responses against tumor and non-tumor antigens in vivo. If so, our model will enable novel ways to investigate tumor immunology of early systemic cancer.

## Supporting Information

Figure S1 **NSG mice engrafted with BM-HSPCs isolated at the day of arrival or the next day.** Adult NSG mice were sublethally irradiated and transplanted with BM-HSPCs that were isolated and transplanted on the day of arrival (immediate, n = 7, 7 donors) or the next day (next day, n = 7, 6 donors). At >20 weeks after transplantation mice were analyzed by flow cytometry. Engraftment in BM and spleen was determined by human CD45 expression. The absolute number of hCD45^+^ cells in BM and spleen of the reconstituted mice is shown, each symbol represents an individual animal.
(TIF)

Figure S2 **The HSPC-yield is independent of the carcinoma type.** No correlation between the number of CD34^+^ HSPCs per 10 million BM-MNCs and the respective type of carcinoma (mammary carcinoma (n = 28), lung cancer (n = 29) and prostate carcinoma (n = 14)) is observed.
(TIF)

Figure S3 **The T cell stimulatory capacity of antigen-presenting cells in reconstituted mice.** **(A)** Different numbers of allogeneic cAPC from BM-HSPC reconstituted mice were used to stimulate CFSE-labeled hT cells and proliferation was measured after 5 or 6 days based on the gradual loss of the CFSE-label. **(B)** Different numbers of mature BM-derived and LPS-stimulated dendritic cells (BM-DCs) from non-reconstituted NSG-HLA-A2/HHD mice were used as stimulator cells for murine CD8 T cells from C57BL/6 mice or human peripheral blood CD8 T cells from an HLA-A2 negative healthy donor. All T cells were labeled with CFDA-SE and proliferation was measured after 5 days based on the gradual loss of the CFSE-label.
(TIF)
