# Supplementary figures and images for: Immune Humanization of Immunodeficient Mice Using Diagnostic Bone Marrow Aspirates from Carcinoma Patients
Source: PLoS One. 2014 May 15;9(5):e97860. doi: 10.1371/journal.pone.0097860 (PMC4022674; doi:10.1371/journal.pone.0097860)

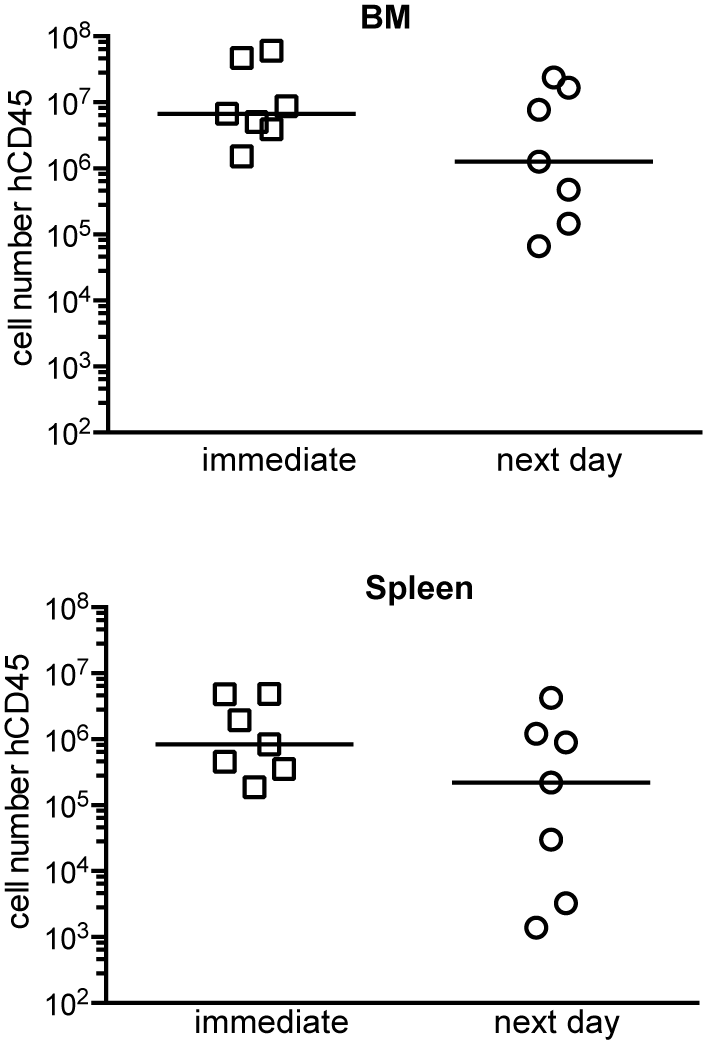

Supplement: Figure S1 — NSG mice engrafted with BM-HSPCs isolated at the day of arrival or the next day. Adult NSG mice were sublethally irradiated and transplanted with BM-HSPCs that were isolated and transplanted on the day of arrival (immediate, n = 7, 7 donors) or the next day (next day, n = 7, 6 donors). At >20 weeks after transplantation mice were analyzed by flow cytometry. Engraftment in BM and spleen was determined by human CD45 expression. The absolute number of hCD45+ cells in BM and spleen of the reconstituted mice is shown, each symbol represents an individual animal. (TIF) [file pone.0097860.s001.tif]

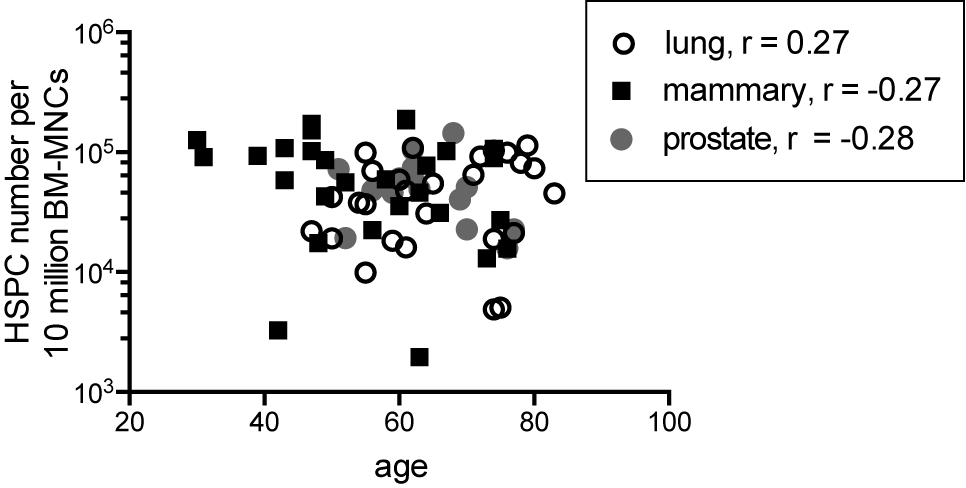

Supplement: Figure S2 — The HSPC-yield is independent of the carcinoma type. No correlation between the number of CD34+ HSPCs per 10 million BM-MNCs and the respective type of carcinoma (mammary carcinoma (n = 28), lung cancer (n = 29) and prostate carcinoma (n = 14)) is observed. (TIF) [file pone.0097860.s002.tif]

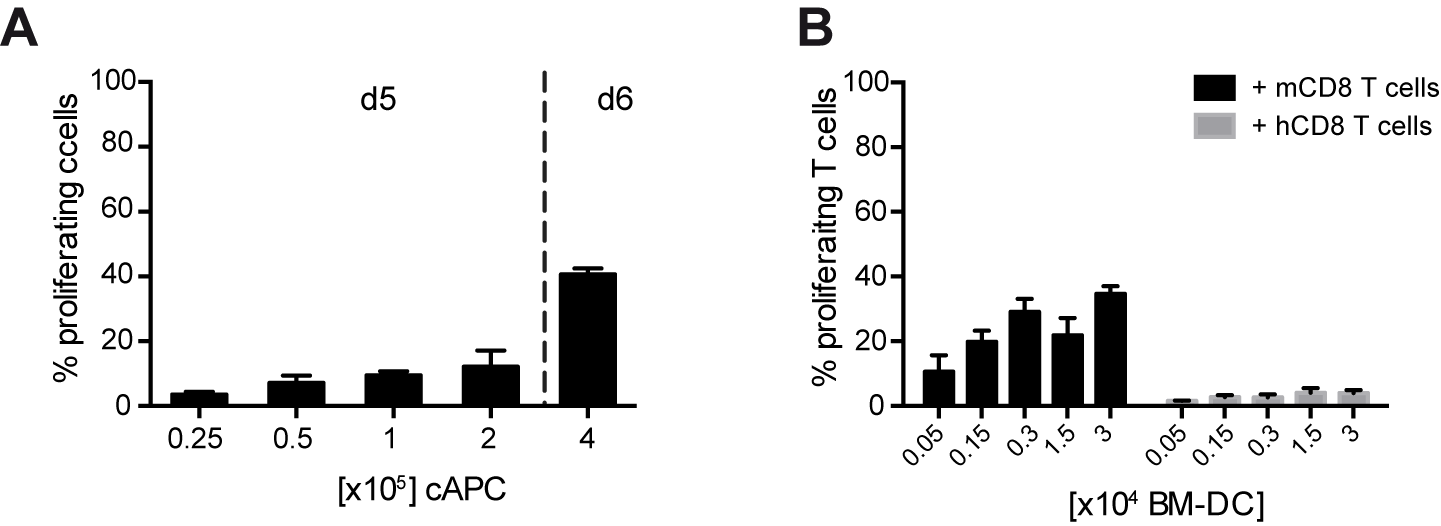

Supplement: Figure S3 — The T cell stimulatory capacity of antigen-presenting cells in reconstituted mice. (A) Different numbers of allogeneic cAPC from BM-HSPC reconstituted mice were used to stimulate CFSE-labeled hT cells and proliferation was measured after 5 or 6 days based on the gradual loss of the CFSE-label. (B) Different numbers of mature BM-derived and LPS-stimulated dendritic cells (BM-DCs) from non-reconstituted NSG-HLA-A2/HHD mice were used as stimulator cells for murine CD8 T cells from C57BL/6 mice or human peripheral blood CD8 T cells from an HLA-A2 negative healthy donor. All T cells were labeled with CFDA-SE and proliferation was measured after 5 days based on the gradual loss of the CFSE-label. (TIF) [file pone.0097860.s003.tif]
